# Sustainable health information exchanges: the role of institutional factors

**DOI:** 10.1186/2045-4015-2-21

**Published:** 2013-05-21

**Authors:** Meir Frankel, David Chinitz, Claudia A Salzberg, Katriel Reichman

**Affiliations:** 1 Emergency Medicine Department, Hadassah Ein Kerem Medical Center, Jerusalem, Israel; 2 Braun School of Public Health, Faculty of Medicine, Hebrew University, Jerusalem, Israel; 3 Bloomberg School of Public Health, Johns Hopkins University, Baltimore, MD, USA

**Keywords:** Health Information Exchange (HIE), Regional Health Information Organization (RHIO), Computer assisted communication, Transition of care, Information flow, Medical informatics, Community, Institutional analysis

## Abstract

The transfer of patient information between the domains of community and hospital influences the quality, continuity and cost of health care. To supply the need for information flow between community and hospital, computerized Health Information Exchange (HIE) systems have evolved. This paper examines the institutional forces that shape HIE development in Israel and in the United States.

In Israel, the vertically integrated Clalit health services developed a different solution for HIE than was developed in the non-vertically integrated Maccabi and Meuhedet health funds. In the United States the fragmented nature of providers – outside of specific networks such as parts of the Kaiser Permanente and Veterans Administration system – have dictated a very different evolution of information flow between community and hospital. More broadly, we consider how institutional factors shape (and will shape) the development of HIEs in different contexts.

This paper applies institutional analysis to explain the emergence of different patterns of development of HIE systems in each of the environments. The institutional analysis in this paper can be used to anticipate the future success or failure of incentives to promote digital information sharing at transition of care.

## Introduction

### Information exchange needed between community and hospital to improve care

Patients with chronic diseases and complex treatment regimens are most at risk when transitioning between hospital and community. Miscommunication or just lack of information may trigger unwarranted and potentially dangerous treatment [1,2]. Discharge from the hospital to community carries significant risk for unintentional medication discontinuation [3].

The paper discharge summary issued to patients when leaving the hospital, like the paper referral letter from a physician in the community, is an attempt to provide information flow between the hospital and the community. In general, traditional paper documents rely on patients to pass on the documents to the community physician. In practice, documents are often not passed on in a timely fashion. Kripalani et al. showed that in 47% of cases, the discharge letter took more than one week to reach the primary care physician in the community [2].

The paper discharge summary suffers from additional deficits. Test results that were not received at the time when the discharge summary is produced will, often, never be seen by the primary care physician in the community. Kripalani et al. reported that at the date of discharge 88% of patients had tests still waiting for results. In addition, paper discharge summaries are not incorporated in the primary care physician’s EMR (if he has one!) and will, effectively, be lost for purposes of follow-up by the primary care physician. In addition, rich patient information (such as the patient’s response to specific drugs) will be lost to the record. This is further complicated by the growing but still limited adoption rate of EMRs by office-based physicians which in the US was only at 57% in 2011 [4].

The paper referral letter from a physician in the community to the hospital is even more problematic. In emergency cases, no letter at all will be available and the admitting hospital will have no reliable record of existing diagnoses, current medications, symptoms or test results from the community. When a paper referral letter is available, the referring physician typically focuses on the immediate reason for referral. In many cases, the referral letter will not include background information. (If the referring physician uses an EMR, the paper referral letter will often include a list of chronic diseases and medications, but typically will not include recent lab results or imaging, when those results are stored in a different computer system.)

Digital information flows have been advocated to address the limitations inherent in paper-based information exchange [5]. In the United States, Regional Health Information Organizations (RHIOs) emerged as an infrastructure, evolving towards Health Information Exchanges (HIEs), in response to the limitations inherent in paper-based information exchange. In Israel, the vertically integrated Clalit Health Services seeks to completely open information exchange between hospital and community care by providing a single medical informatics system across the spectrum of care. In each country, and within each country, institutional factors have shaped and continue to shape the development of health information exchange.

### How institutional factors shape information flow

Often, problems that seem purely technical, such as data flow through a health provider network, are addressed by discrete technical fixes. Ideally, technical solutions to technical challenges will factor in the institutional context that will both influence and be influenced by the introduction of technical interventions. To place health information exchanges in institutional context, we present some factors to consider in planning, as well as simulating what is likely to happen in the implementation process [6].

The arrangement of healthcare institutions in a society or geographic unit will directly affect the factors that influence the rate and cost of adopting health information exchange between partners in a coalition. These factors include the level of centralization of decision making in the coalition, the starting point for electronic medical records, the number/diversity of records systems that need to be integrated into the exchange, and the scale (how many records).

Despite seeming to be an obvious consideration, it is often overlooked that introduction of Health Information Technology (HIT), not to mention attempts at inter-organizational transfer of data, will be impacted by the status of such systems at the outset of policy intervention. In organizations and health systems with strong histories of HIT development and utilization, information transfer issues will play out differently than in systems with nascent HIT systems. On one hand, a tradition of information technology at the individual provider – for example, health plan or hospital – level can serve as an infrastructure which facilitates information transfer. On the other hand, organizational level weddedness to existing systems, as well as proprietary and political interests may block smooth integration of systems across organizations. Alternatively, systems seeking to introduce both new information systems and guarantee transferability of data may be biting off more than they can chew.

Rate and cost of HIE adoption is also a function of the starting point: the level of computerization within each of the partners and between the partners in a network before foundation of the HIE. In a case where some members of the network have no electronic medical records, HIE implementation will be delayed until the member organizations adopt computerized systems. In Israel, the nature of affiliation between community physicians and the Health Maintenance Organizations (HMOs) enabled the HMOs to require almost totally electronic records for community care. In the United States where the relationship between payers and providers is much looser, in general, individual practices and hospitals have been slower to adopt electronic records [7,8]. Related to history, starting point, and level of computerization is culture. In cultures where there is an overall push to technology and information systems, especially if physicians are part and parcel of such a culture, implementation of HIT will be enhanced. In such a culture, movement towards health information exchange may be driven at the grass roots as part of an overall societal orientation towards computerization. Where cultural factors work against HIT (for whatever reason, such as professional status or working conditions of physicians, the size of physician practices [9], or issues of privacy) and medical culture has been slow to adopt information technology, policies directed at development of health information exchanges and their integration face cultural as well as technical challenges.

The institutional linkages among organizations will also have an impact. All information exchange solutions carry costs, startup implementation costs and ongoing transaction costs. Transaction costs will be higher, and rate of adoption will be slower, in loosely grouped coalitions of providers. An institutional arrangement where one overarching framework is available to allocate funds for paying costs and mandate implementation will move faster than an institutional arrangement with many completely independent actors [10]. For example, the United States Department of Veterans Affairs (VA) was an early adopter of health information flow within their extensive network thanks to highly centralized management.

HIE adoption costs will also be a function of the number of local systems that need to be integrated. In Israel, each of the HMOs has implemented standards-based records for community care across the network of providers. In the United States, individual practices who have adopted a software system have chosen from a plethora of available systems, making integration more expensive and more difficult.

The scale of institutions affects the unit cost for information exchange systems. In Israel, with relatively large HMOs covering millions of subscribers, information exchange systems within each HMO were adopted relatively early and did not require government subsidies or regulations. In the United States, with many independent physicians and many small groups of physicians, the unit cost has been prohibitive and – prior to the era of Accountable Care Organizations (ACOs) – HIEs have withered as soon as government or philanthropic subsidies for the exchanges became exhausted.

Finally, the contingencies of national policy regarding health information systems will influence the type of systems that emerge, the incentives of individual organizations and players to cooperate, and the smoothness of implementation.

In the remainder of this paper, we employ this institutional lens in a comparative context, discussing the evolution of health information systems, in particular attempts at information transfer, in the US and Israel.

### The response to the need for information flow in the United States

The state and prognosis of health information exchange in the United States is a direct function of institutional and other non-technical factors, including factors that are unique to the American experience.

### Developing standards to enable information flow

When different providers operate different medical record software need to exchange data, ideally each software system would adopt a single standard to enable interoperability. In fact, multiple standards compete for the position of “the” standard, such as the Health Level 7 (HL7) Clinical Document Architecture (CDA) and The American Society for Testing and Materials (ASTM International) Continuity of Care Record (CCR) [11]. Even when a standard is adopted, differing implementations of the standard pose ongoing interoperability challenges [12].

Standards alone have not been sufficient to promote widespread health information flow. Institutional factors and economic incentives have been key players in those cases where health information is shared between providers.

### The development of regional health information organizations (RHIOs)

Prior to 2010, the focus of the Nationwide Health Information Network (NwHIN) [13] was on facilitating Regional Health Information Organizations (RHIOs). A RHIO seeks to provide health information exchange between health care stakeholders within a defined geographic area. The goal was to improve care, but the broad regional sweep of RHIOs did not factor in the inherent institutional relationships between the providers.

The RHIOs had critical challenges, including compensating for the American resistance to using a unique identifier for people and dealing with a fragmented health care delivery system, even within small geographical area (Table 1).

**Table 1:** Critical Tasks for RHIOs

**Task**	**Description**	**Why needed**
Identifying who’s who	Sifting through different medical record numbers used by different medical providers and health systems to determine what records belong to a patient.	Resistance to using a unique universal identifier.
Melding disparate records into a coherent picture	Combine medical records for a particular patient across disparate providers and present an intelligible picture for the clinician.	Health providers over spectrum of community and hospital are fragmented without a common medical record and without common data standards.

RHIOs, initially supported by grants, were supposed to become self-supporting by charging fees to providers and insurers. However, starting in 2010, before many RHIOs became self-supporting, the Office of the National Coordinator (ONC) has been sponsoring policies that promote Health Information Exchanges (HIEs), rather than RHIOs. Grant funding for RHIOs has largely ended, forcing RHIOs to rely on fees from providers and insurers [14]. As demonstrated by Adler-Milstein et al. [15], early dependence on grants is associated with a lower probability that a RHIO will be operational or financially viable.

In general, the RHIOs do not have a sustainable business model and are probably not viable without ongoing government funding. The geographical focus of RHIOs carried no inherent business incentives for providers and insurers. The costs of information exchange within an RHIO are not tied to possible gains, because the members in the organization do not share profits and losses. With government support under the Bush administration, the number of RHIOs expanded between 2005 and 2008 to over 100 active organizations. Even under this period, however, achievements of the RHIOs were limited because of inadequate funding. Under the Obama administration, support for RHIOs has been reduced and RHIOs have shrunk. By December 2009, only 75 RHIOs were operational with coverage of approximately 14% of U.S. hospitals and only 3% of ambulatory practices. Of the 75 operational RHIOs, only 25 met criteria for financial viability, only thirteen RHIOs supported stage 1 meaningful use and none met an expert-derived definition of a comprehensive RHIO [16].

While RHIOs may be useful in improving information flows between providers, and may improve patient care, institutional factors were not aligned to provide viable ongoing sustenance for RHIO. Essentially, RHIOs are on artificial life support, connected to government or philanthropic largesse and when the flow of support is cut off, the RHIO shuts down.

The Indiana Network for Patient Care (INPC), a city-wide clinical informatics network in Indianapolis, is an excellent example of a RHIO that works, and illustrates how the RHIO is dependent on external funding. The INPC is substantially dependent on funding from the Regenstrief Institute. (The Institute is named for Samuel N. Regenstrief, an industrial production expert who invented the low cost front-loading dishwasher.) Without funding from the Regenstrief Institute, the INPC would not be able to present information about patients as one virtual medical record regardless of where they received prior care.

### The development of health information exchanges (HIEs)

**Figure 1:**
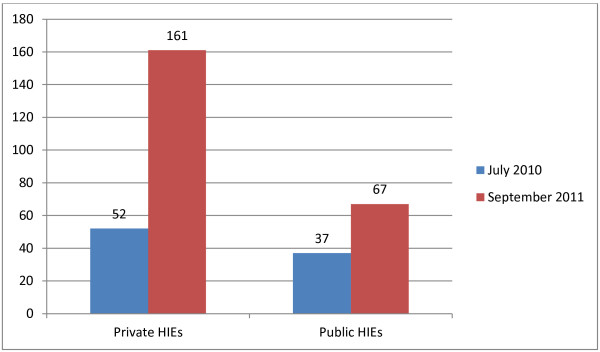
**Number of "Live" HIEs (data from: *Sustainable Success: State CIOs and Health Information Exchange*) **[18]**.**

In the United States, with the passage of the Health Information Technology for Economic and Clinical Health (HITECH) Act, enacted as part of the American Recovery and Reinvestment Act of 2009 under the Obama administration, Health Information Exchanges (HIEs) emerged as a response to the need for better information flow. In particular, the HITECH Act allocates US$564 million for state-level health information exchanges [17]. HIEs facilitate the movement of actionable health care information within or across organizations. HIEs owe much in terms of computing standards and standardized medical vocabulary to RHIOs, but HIEs have a different business model and focus. Some HIEs, like RHIOs, are state-sponsored, but HIEs can also be private profit or not-for-profit organizations. The number of private HIEs increased by more than three times over a fifteen month period and now exceeds the number of public HIEs by 2.4 times [18] (Figure 1).

While RHIOs were typically focused on state or municipal boundaries, privately-sponsored HIEs are likely to draw patient data across population catchment areas and will pay less attention to state or municipal boundaries. (Like RHIOs, state-sponsored HIEs may tend to follow state boundary lines. Private HIEs will follow coalitions of providers under institutional umbrellas, such as an ACO).

The emergence of HIEs has been driven by push factors (business need to get and keep referrals and government incentives for meaningful use), has been enabled by the emergence of lower cost distributed computing solutions, and – like RHIOs – have been promoted by government grants. The divergence of government grants from RHIOs to HIEs effectively promotes HIEs in most areas of the country.

As shown in Figure 1, the private sector has accelerated adoption of HIEs, with the number of private HIEs increasing by more than 200% over 15 months while public HIEs increased by 80% during the same time period.

### Referrals as a business driver for HIEs

HIEs carry the promise of improved care, but also provide strong business incentives for adoption. The motivation for adoption of HIEs by private operators is increasing/maintaining patient volume. Providers who cooperate in the HIE hope to increase referrals within the network of partners in the HIE, driving patients to specialists, outpatient facilities and hospitals in the HIE network [19]. For practice groups, and affiliate networks of hospitals and physicians, keeping referrals within their networks is a prime business goal. In the US, almost sixty percent of patients seeking a specialist base their decision exclusively on physician referrals and nearly three in four undergoing a procedure choose the hospital based on a physician recommendation or referral [20]. Whoever can share referral information and follow up with primary care physicians will win and keep referrals.

The shape of networks in different locations will drive demand for record sharing via a HIE. A hospital that owns a large network of community physicians and requires all physicians to use the same EMR would have less need for a HIE. But a similar hospital with fewer community physicians on the same EMR would have a greater need for a HIE.

As described in the Bay Area case study (next page), the most important driver for major players adopting the HIE was the ability to generate referrals.

### Government incentives drive HIEs

HIEs, and the interoperability standards needed to support HIEs, are promoted by Medicare and Medicaid health IT incentives for healthcare organizations to achieve “meaningful use” of EHRs. Between 2011 and 2015, American Recovery and Reinvestment Act of 2009 (ARRA) incentives may total as much as $48,400 for eligible professionals and up to $11 million for hospitals.

HIEs dependent on government incentives will always be vulnerable to changes in government regulations. Hospitals and community providers, aware of the fickle nature of government incentives and rules, will be reluctant to make the needed investments under conditions of uncertainty. Should the prevailing attitudes among policy makers shift – as was the case for RHIOs with the shift to the Obama administration – the move to HIEs will lose momentum, for those HIEs with insufficient business incentives.

### Government grants drive HIEs

In addition to business drivers and meaningful use incentives, the Health Information Exchange Cooperative Agreement Program alone has distributed nearly $550 million dollars in funding. As of March 2010, the number of State HIE Cooperative Agreement Program awardees totaled 56. (The number of awardees explains the jump in the number of state-run HIEs between July 2010 and September 2011, as shown in Figure 1.)

As noted in the *Sustainable Success: State CIOs and Health Information Exchange* report, one of the conditions for continuing awards is that the HIE can demonstrate that it will generate enough revenue to cover all the costs for a functioning HIE [18].

HIEs dependent on government grants will always be vulnerable to changes in the availability of grants. Since the HIE is, in some sense, a public good, it’s not clear at all that state-run HIEs will develop viable sustainable business models to keep HIEs running in the absence of federal largesse. The experience of RHIOs indicates that making the move to a subscription fee or usage fee model for HIEs will not be easy. The preponderance of private HIEs is encouraging because private HIEs already have an institutional framework for budgeting and allocations. To the extent that the business case for information exchange is compelling, the private HIE can leverage their existing budget framework to support ongoing operations without depending on marketing fee for service programs to providers who participate in the information exchange network.

### Case study: the San Francisco Bay area HIE

The San Francisco Bay Area HIE covers more than 2,800 physicians and over 900,000 patients (Table 2). An important motivator for one of the key players, UCSF, was the lack of communications between referring physicians and staff physicians at UCSF. As noted (see “Referral as a business Driver for HIEs” above), medical centers such as UCSF are highly dependent on physician referral. In fact, the first two features that UCSF implemented were test result sharing and secure messaging with patients and referring physicians. The combination of these features enables UCSF physicians to review results and discuss their findings with patients and/or referring physicians [19]. In this case, for UCSF, the need to maintain and expand the flow of referrals was the critical motivating factor for HIE adoption.

**Table 2:** The players in the bay area HIE

**Player**	**Type**	**Description**
University of California, San Francisco (UCSF)	Hospital	600-bed tertiary and quaternary regional referral medical center. Averaged 740,000 ambulatory visits in 2008.
John Muir Health	Hospital + Physician Network	Not for profit. Includes two medical centers (581 beds), a physicians network and outpatient clinics.
Hill Physicians Medical Group	IPA	Large IPA provides primary and specialized care to nearly 300,000 people.
Alta Bates Medical Group	IPA	600-physician IPA. Serves 50,000 people.
San Ramon Regional Medical Center	Hospital	123-bed acute care hospital.

Note: IPA, Independent Physicians Association; The Bay Area HIE is one of the HIEs in California.

### ACOs will affect demand for information flow

An Accountable Care Organization (ACO) is a legal entity composed of a network of doctors and hospitals that share responsibility for providing care to patients. The ACO is a provision of the Accountable Care Act intended to bring down costs in the Medicare program (ACA section 3022). Under current legislation, an ACO would agree to manage all of the health care needs of a minimum of 5,000 Medicare beneficiaries for at least three years.

To win and keep customers, ACOs will need to prove that the overall health care product they deliver works better. To make profits, ACOs will need to be efficient low-cost providers. Unlike traditional fee for service Medicare payments, ACOs incentivize providers need to reduce costs (Table 3). In this aspect, ACOs resemble HMOs in Israel who receive capitation fees and have strong incentives to use technology to reduce costs.

**Table 3:** How ACO incentives influence providers

**Fee for service under Medicare**	**Incentive payments for ACOs**
Fee-for-service payments. In general, doctors and hospitals are paid more when they give patients more tests and do more procedures.	Bonuses when ACOs keep costs down and meet specific quality benchmarks, focusing on prevention and carefully managing patients with chronic diseases. Providers get paid more for keeping patients healthy and out of the hospital.

While physicians will likely want to refer patients to hospitals and specialists within the ACO network, patients will be free to see doctors of their choice outside the network without paying more. Unlike in an HMO, an ACO patient is not required to stay in the network. As a result, physicians will need to balance patient satisfaction with low-cost per patient. Use of an HIE to maintain information flow across the provider spectrum within the ACO contributes to both goals: patient satisfaction and low-cost operation [21].

To reduce costs over a multi-year period, ACOs have to make up-front investments in improving care (such as adding case managers) *and* in better coordinating care between different providers in the community and in hospitals. Care coordination requires two-way communication between community and hospital providers. For this reason, we anticipate that a shift to ACOs will accelerate the movement towards HIE and will promote improved information flow at the transition of care between community and hospitals.

The advantage of large numbers of patients for players in the ACO market will encourage growth of large multispecialty physician groups and will encourage hospital systems to buy up physician practices. The larger the grouping, and the greater the vertical integration of the providers from physicians in the community to hospitals, the easier it becomes to provide a single Electronic Health Record (EHR) with information that flows across traditional boundaries.

### How medication reconciliation programs will affect information flow

Medication reconciliation programs are endorsed by the Institute for Healthcare Improvement, are part of the United States’ Joint Commission’s National Patient Safety Goals, and are part of the “meaningful use” criteria under the American Recovery and Reinvestment Act. As the Centers for Medicare and Medicaid Services Process of Care (Core) Measures move from a pay-for-reporting structure to reimbursement based on performance (value-based purchasing) [22], hospitals will seek means for achieving medication reconciliation. Since it will not be feasible to automatically get medication information from the community, or to send medication information from the hospital to the community, without use of HIEs, incentives for medication reconciliation will drive hospital demand for participation in HIEs.

## The response to the need for information flow in Israel

In Israel, universal national health insurance was legislated in 1994. There are 4 HMOs: Clalit, Meuhedet, Maccabi and Leumit. The HMOs are both the insurer and the provider of at least primary care in the community. The citizen pays health taxes according to income, and can choose freely between the HMOs without regard to their income and without limitation based on their health status.

The HMO differ in structure (how primary care physicians are compensated/employed), size (number of patients), and scope. All of the HMOs offer networks of primary care physicians, at least some secondary care, and operate laboratories. The HMOs operate at least basic radiology services (X-ray, ultrasound) with supplementary imaging and diagnostics provided by independent imaging centers or imaging centers in hospitals. In some areas, HMOs operate day-care units. Clalit is the only HMO to operate a network of hospitals, including some of the most advanced and largest hospitals in Israel. (Maccabi operates a profit oriented hospital that does not provide emergency or many other services.) Clalit makes use of other hospitals, especially in areas where there is no Clalit hospital (as in Jerusalem). The other HMOs direct their patients to Clalit or non-Clalit hospitals based on location, specialty and the financial arrangements that each HMO negotiates with each hospital.

**Figure 2:**
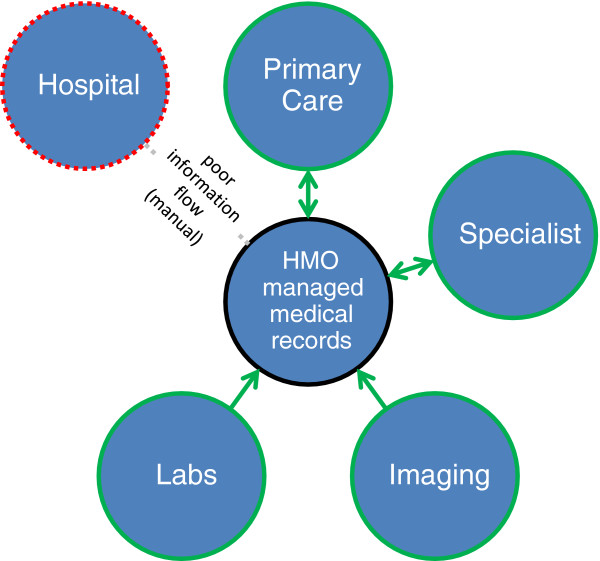
Current State of Information Flow Hospital <-> Community (outside of the Clalit HMO).

This situation where patients cross organizational lines, with manual information flow between hospital and community, leads to transition of care problems. Patient medical records, traditionally, existed in a separate silo for each HMO, with additional silos in specific hospitals where the patient may have been hospitalized. When a patient is admitted, the physician at the hospital has to get the medical information from the patient himself or from a referral letter (if available). Information flow stops at institutional boundaries (Figure 2).

Unlike in the United States, in Israel virtually all community care records are computerized in a single system for each HMO. In addition, patient records in Israeli hospitals are – for the most part – fully electronic. Within the HMO, physicians – and patients – have electronic access to a range of medical records including lab results and imaging. In the United States, a move towards Health Information Exchanges (HIEs) may first require universal adoption of electronic medical records in community care and in hospitals. In comparison, HIE implementation in Israel starts from fully functioning hospital systems and comprehensive in-place community medical records.

### Government and HMO influences on information exchange evolution

In 2004 the Ministry of Health decided to establish a country-wide HIE, the National Electronic Medical Registry (NEMR). Using the proposed NEMR, any admitted hospital could get critical information from other hospitals and HMOs, including lists of chronic diseases, allergies and chronic medications. The NEMR project has not come to fruition yet and, to the best of our knowledge, has been effectively stalled for approximately eight years. Even should the NEMR move ahead, the NEMR as currently envisioned would not provide full two-way information flow. The NEMR would not address the need to share hospital information with community caregivers.

In the vacuum left by the absence of a top-down NEMR, the HMOs and the hospitals established, separately, HIEs. Initially, the HIEs were restricted to vertical integration within a single provider. Gradually, the HIEs began to cross institutional boundaries. The pioneer in cross-institution medical record exchange was the OFEK system, established by Clalit. Initially, OFEK bridged community and hospital care within the Clalit network. The Ministry of Health widened the scope of exchange by funding connection of government owned facilities to OFEK. Later, Hadassah Medical Center connected to the Maccabi and Meuhedet HIEs.

### The case of the vertically integrated Clalit Health Services

**Figure 3:**
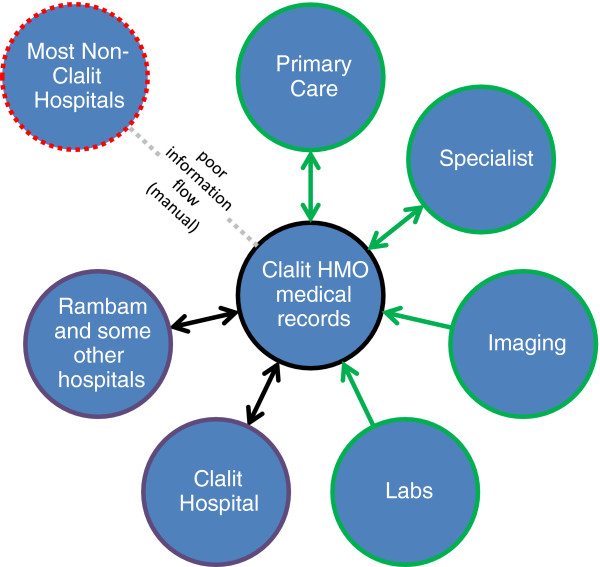
Information Flow Hospital <-> Community within Clalit.

The Clalit HMO has the full spectrum of health services from primary care through tertiary medical. To improve the information flow within the network of Clalit community care and hospitals, in 2005 Clalit launched an innovative system of hospital-community on-line medical records called OFEK (a dbMotion software product). Through the OFEK system, all Clalit clinics and hospitals can communicate and get information on all the patients in Clalit (Figure 3).

In addition to the Clalit hospitals, three government-owned hospitals joined the OFEK system: Rambam, Sheba and Wolfson. Rambam and Sheba are major tertiary care centers. Currently, there is no connection between OFEK and other non-Clalit HMOs. Nirel et al. found that “at clinics in catchment areas of hospitals using OFEK extensively, OFEK reduced the number of imaging tests and, to a lesser extent, laboratory testing and improved several quality measures” [23].

Unlike physicians in other HMOs, Clalit physicians uniquely have universal access to records for their patients everywhere – in the community and in Clalit hospitals. When Clalit embarked on the OFEK project the other HMOs led the field in sharing medical records across the community. With OFEK, Clalit has been able to deliver a single solution for both community and hospital records.

The vertical integration across hospital and community care explains why Clalit has been a pioneer in sharing electronic health information across the hospital-community divide.

### The case of Hadassah hospital and the HMOs: records crossing borders

**Figure 4:**
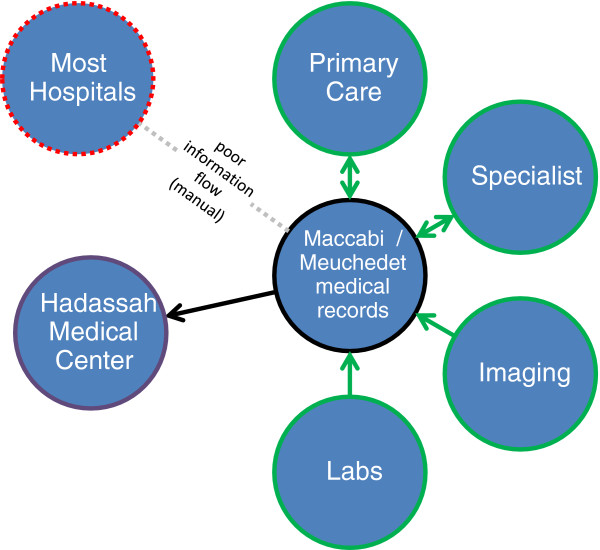
Information Flow Hospital <-> Community at Hadassah.

Hadassah Medical Center is a non-profit independent organization which operates two hospitals in Jerusalem - Hadassah Ein Kerem Hospital and Hadassah Mount Scopus Hospital (1000 beds). Hadassah is a tertiary medical center that serves patients from all over the world but mainly from Jerusalem. All four HMOs direct their patients to Hadassah as well as to other hospitals in Jerusalem. In order to improve the connection between Hadassah and the HMOs, Hadassah established a computerized-connection between Hadassah and two HMOs: Maccabi (2009) and Meuhedet (2011). This connection enables hospital physicians to see critical details from the patient’s electronic record in the HMO (including chronic medications, lab results, and imaging results) (Figure 4). For privacy reasons, hospital physicians can only access information about the patient while the patient is hospitalized in Hadassah. This is a unique project, enabled by physicians and medical informatics experts from the hospital and the HMOs, dedicated to improved quality of care. The use of the computerized connection is growing, with more than 1,000 entries a month, especially after an “academic detailing” intervention [24].

The HMOs have an incentive to invest in improved information flow with Hadassah more than in hospitals in other cities because of the absence of a Clalit hospital in Jerusalem. However, the same competition for patients, and the same potential gains from improved transition of care for patients transitioning between hospitals and community, will drive the HMOs to seek similar arrangements with other hospitals and in other cities. For Hadassah, the desire to earn more referrals from community physicians is an incentive to invest in the system, as well as a desire to leverage information flow to provide the safest, most optimal care. In addition, we attribute success to a fortuitous combination of key personnel involved at Hadassah, Maccabi and Meuhedet. Planning and implementation were supported by strong lobbying on the part of staff physicians, managers and the Hadassah Center for Clinical Quality and Safety.

### Using institutional analysis to understand and recommend

The institutional analysis in this paper can be used to explain and to anticipate the success or failure of digital information sharing at transition of care, as well as the course of developments in the evolution of health information exchanges.

### Understanding past developments

In the US and Israel, the nature and direction of information flow at the transition of care between hospital and community can be understood in the context of the institutional relationships between providers in the community, and between hospital and community providers (Table 4). Consistently, the presence and quality of computerized information flow system between providers, especially between hospital and community, is a function of the tightness of the relationship between the providers.

**Table 4:** Factors influencing information at the transition of care

**Factor**	**Influence on Information Flow**
Fragmented relationships in the United States between providers (outside of the VA, Kaiser and additional exceptions)	An extremely fragmented relationship between providers in much of the United States has meant almost no electronic information flow within and between primary, secondary and tertiary providers. When government agencies provide carrots and sticks to encourage information flow, RHIOs and HIEs emerge. When government funding is removed – as for the RHIOs –the absence of coherent sets of providers/insurers with financial incentives to make the exchanges work led, in most cases, to the closure of the exchanges.
Clalit HMO: a vertically integrated health service provider across primary, secondary and tertiary care	A fully vertically integrated health service provider that covers the full spectrum of needs for hospital and community, Clalit, has excellent information flow between hospital and community.
Maccabi, Meuhedet and Leumit HMOs: integration in primary and secondary care only	Health services providers that provide integrated community care – the Maccabi, Meuhedet and Leumit HMOs in Israel – have excellent information flow between providers in the community, but the information flow breaks down when patients transition in or out of the hospital (with an important exception for Maccabi, Meuhedet and Hadassah).

**Table 5:** Forecasts and recommendations

**Environment**	**Forecasts**	**Recommendation (to improve transition of care)**
United States: HIEs	• Changes in administration funding priorities that reduce government support for HIEs could seriously threaten the move to HIEs.	• Provide interim funding for successful HIEs (in terms of volume of use) while making clear the timelines for a shift to private financing.
	• There will be a “shake out” as some HIEs discover that they do not have a viable business model.	• Continue move to incentives for value-based purchasing. This will encourage providers to invest in development and support of solutions that improve information flow at transition of care.
	• Shift to ACOs will tend to accelerate growth of HIEs.	
	• Changes in reimbursement rules are likely to continue the move towards vertical integration of providers (hospitals acquiring group practices). Vertical integration will facilitate improved information flow.	• Publish and support standards for coding medical information to facilitate structured vertical data sharing between different providers.
		• Avoid the temptation to seek maximum data sharing that is not focused on providing benefits to ACOs. Seeking a maximal goal of totally free data flow between providers could undercut the financial incentives driving providers to join HIEs and share data.
		• Eliminate incentives to not modify medication upon discharge (avoid perverse effect of medication reconciliation programs).
Israel: information flow at hospital intake/discharge	• The non-Clalit health care providers will piggyback on OFEK, extending the reach of OFEK to the other HMOs, to government funded and other non-Clalit hospitals, and to other rehabilitation centers and extended care facilities.	• Support easier information flow with promotion of standardized vocabulary/guidelines between the various HMOs and hospitals.
	• The vertical integration of the HMOs provides strong ongoing incentive for improving information flow at hospital intake/discharge. We anticipate that information flow will continue to improve, either through improvements to OFEK or through development of new systems.	• Mandate information sharing.
		• Mandate improvements over time, such as alarms for test results received after the date of discharge. Mandated improvements will drive future enhancements to OFEK or other HIE systems.
	• Clalit, by virtue of its ownership of hospitals, completing the medical services supply chain, and by virtue of its size, is likely to remain a dominant force in HIE and may continue to set de facto standards and platforms for information flow.	

In this context, at first glance the information flow from Maccabi and Meuhedet to Hadassah seems somewhat of an outlier. In addition to improvements in the quality of care, the incentives for Maccabi and Meuhedet to invest in the system are the hoped for gains from improved transition of care for patients transitioning to one of the Hadassah hospitals. Leumit is not involved, at least not yet, because its smaller size will yield a smaller payoff. Hadassah’s incentive to invest is to earn more referrals from community physicians (the assumption is that improved information flow will increase referrals). In addition, we attribute Hadassah’s willingness to invest to strong lobbying by staff physicians involved with the Hadassah Center for Clinical Quality and Safety [25].

### Future developments and policy recommendations

Building on our institutional analysis we can anticipate the success or failure of incentives, and can offer recommendations to shape the evolution of digital information sharing at transition of care in different environments. For both Israel and the US, a key insight is that single-minded focus on the technical and logistical challenges of implementing Health Information Exchanges will miss the important market structure, legal and cultural contingencies that are as important. Moreover, a top-down orientation, by which national government seeks to promote deployment of HIT may not only run afoul of local contingencies, but also miss out on the potential of grass roots initiatives. Too often, it seems that well intentioned pushes for using HIT to, for example, reduce administrative costs, fail to give adequate attention to such considerations (see, for example, Cutler et al. 2012) [26].

Regarding the United States, where fragmentation of providers along the spectrum of care and separation between providers and payers is the norm, we do not expect information flow at transition of care between community and hospital environments to evolve organically. While external incentives such as grants may stimulate development of information exchanges, sustainability in information exchanges beyond a grant period requires structural changes that will increase the payoff from sharing information. The single most important change that we anticipate is change in reimbursement policy – such as the ACOs – that provide payoffs sufficient for providers to cooperate in information flow. The question will be whether ACOs can create the institutional conditions for collaboration among erstwhile separate providers in order to justify, from a cost point of view, the development and maintenance of information exchanges. This is crucial, as government and private grant support will be insufficient to create long-term sustainable HIEs (Table 5).

In addition to government grants and incentives, private sector funds (such as those provided by the Regenstrief Institute) and insurance rules can trigger development and promote adoption of solutions that facilitate information flow at the transition of care. However, fragmentation of providers means that solutions in the United States will be more difficult to implement, more costly to develop, and more expensive to maintain over time. The policy implication is that substantial ongoing incentives will be needed to create and sustain HIE systems over time. ACOs are an example of one instance where structural change provides incentives to promote and support information exchange. ACOs will, we anticipate, lead to development of sustainable HIE, albeit within the confines of each ACO. The Veterans Health Administration (VHA) provides an instructive example of how institutional factors can support information flow. The integrated nature of the VHA, the long term view that the VHA adopted, as well as the VHA drive for quality, have all contributed to successful health information exchange within the VHA.

In Israel, where community health records are fully computerized for all four HMOs, it seems that computerized information flow will evolve faster, more effectively and relatively less expensively – at least in the near term – than health information exchange in the US, where the starting point for community records is less integrated. The health services in Israel will continue to bridge the divide separating hospital and community care, but in the future will do so less by developing ad hoc solutions for each HMO based on linking each HMO to medical records in individual hospitals or hospital networks. Instead, the divide separating hospital and community care will be, increasingly, bridged by the expansion of OFEK to other non-Clalit hospitals. OFEK will be used for all HMOs to share information with hospitals. After OFEK implantation becomes universal, there will be institutional pressure to expand the functionality of OFEK. For example, we expect the HMOs, including Clalit, to expand OFEK to deliver alerts and warning for information from hospital information to community care. If OFEK does not respond to institutional pressures, we would expect the reemergence of various ad hoc hospital-by-hospital direct information exchange between HMOs and individual hospitals (similar to the arrangement already up and running for Meuhedet and Maccabi with Hadassah).

## Conclusion

In this institutional analysis we described the development of RHIO and HIE systems in the United States and in Israel. We detailed the multi-layered relationships between the health institution structure and the process of development health information exchange between different community and hospital health providers.

Worldwide, HIE development is key to improving quality of care and containing costs. The very different experiences of the United States and Israel provide lessons that can be applied in different countries with very different institutional arrangements for health care.

Before trying to develop or improve an HIE system in any country, health policy authorities should consider how local health institution structures will affect the main players in the field. Understanding the whole ecosystem of institutional relationships, incentives and interests will aid decision makers in formulating policies that will be effective for optimal HIE development and implementation.

## Competing interests

MF is a salaried employee in Hadassah Medical Center & Clalit Health Services. DC is a salaried employee in Hebrew University. CS is a PhD student in Johns Hopkins University. KR is a self-employed software engineer. None of the authors got funding for this research paper.

## Authors’ contributions

MF collected, analyzed and summarized the information on computerized HIE between hospitals and community in Israel, and read and approved the final manuscript. DC proposed the initial idea of the paper, made critical revisions, and read and approved the final manuscript. CS made critical revisions and wrote part of the section on HIE in the US. KR analyzed and summarized the information on RHIOs and HIEs in the US, and wrote, read and approved the final manuscript.

## Authors’ information

MF is a senior physician in the Emergency Medicine Department in Hadassah Ein Kerem Hospital and practices internal medicine at a Clalit Health Services clinic in Jerusalem. DC is a Professor of health policy and management in the Braun School of Public Health, Hebrew University, Jerusalem. CS is a PhD student in the Department of Health Policy and Management of the Bloomberg School of Public Health, Johns Hopkins University, Baltimore, MD, USA. KR is a medical informatics specialist and a PhD candidate in the Braun School of Public Health, Hebrew University, Jerusalem.
